# Sustained intrathecal delivery of amphotericin B using an injectable and biodegradable thermogel

**DOI:** 10.1080/10717544.2021.1892242

**Published:** 2021-03-04

**Authors:** Wenting Lin, Tao Xu, Zhongzhi Wang, Jianghan Chen

**Affiliations:** a Department of Dermatology, Changzheng Hospital, Naval Medical University, Shanghai, China; b Institute of Internal Dermatology, Shanghai Skin Disease Hospital, Tongji University, Shanghai, China

**Keywords:** Amphotericin B, PLGA–PEG–PLGA, intrathecal injection, cryptococcal meningitis, sustained release

## Abstract

Cryptococcal meningitis is a fungal infectious disease with a poor prognosis and high mortality. Amphotericin B (AMB) is the first choice for the treatment of cryptococcal meninges. The blood-brain barrier (BBB) is the major barrier for the effective delivery of drugs to the brain. In this study, AMB was incorporated in a thermosensitive gel for intrathecal injection. We first synthesized AMB-loaded thermogel, investigated its in vitro cumulative release, and in vivo neurotoxicity, and therapeutic effect. The thermosensitive gel was comprised of 25 wt% poly (lactic acid-co-glycolic acid)-poly (ethylene glycol)-poly (lactic acid-co-glycolic acid) (PLGA-PEG-PLGA) triblock polymer aqueous solution. The AMB loaded in the thermosensitive gel (AMB in gel) had low viscosity at low temperature and resulted in the formation of a non-flowing gel at 37 °C (physiological temperature). AMB loading in gel sustained its release for 36 days and the *in vitro* cumulative release rate was satisfactory. Compared with the AMB solution, intrathecal administration of AMB in gel could reduce the neurovirulence of AMB and get a better treatment effect. The findings of the current study show that the injectable PLGA–PEG–PLGA thermogel is a biocompatible carrier for the delivery of drugs into the intrathecal.

## 1. Introduction

Cryptococcal meningitis (CM), an opportunistic fungal infection caused by Cryptococcus neoformans or *Cryptococcus gattii*, is a fatal central nervous system (CNS) disease with a worldwide distribution. CM is the leading adult meningitis in areas with a high incidence of HIV (Rajasingham et al., 2017; Williamson et al., 2017). Besides, it also shows a growing number of immunocompromised and immunocompetent patients. In the USA, the death rate in HIV-negative CM individuals was around one quarter of CM-associated hospitalizations and one-third of CM-associated mortalities (Perfect et al., 2010; Williamson et al., 2017; Migone et al., 2018).

AMB, a member of polyenes, is the first-recommended drug for the treatment of CM (Williamson et al., 2017). The pharmacokinetics studies have shown its poor penetration into the cerebrospinal fluid (CSF) and brain parenchyma due to its molecule properties (Hamill, 2013) and blood-brain barrier (BBB). BBB is an impermeable cellular interface that separates the blood from the brain, selectively allows only a few substances to pass. The concentration of AMB in CSF was approximately 0.05 mg/L following its intravenous injection, 10 times lower than used for antifungal activity (Nau et al., 2020). Besides, its intravenous administration has many side effects such as headache, fever, damage of liver and kidney, and vasculitis, etc (Nau et al., 2020)., which makes it far from being the best choice.

New therapeutic approaches including intrathecal injection increase the concentration of AMB in CSF. However, they are required to be performed by lumbar puncture 2–3 times a week with inevitable neurological toxicity symptoms like headache, neck stiffness, lower limb pain, urinary retention, and even lower-limb paralysis. Intraventricular infusion with Ommaya reservoirs (Nakama et al., 2015) and lumbar puncture drainage (Yuchong et al., 2011; Fang et al., 2012) of AMB has been reported to control CM effectively, a 1-h intrathecal infusion rather than injection could reduce neurotoxicity, however, it is easy to cause superinfection and more nursing care is needed. The ideal therapeutic drugs are supposed to have a high concentration in the brain tissue or CSF and fewer side effects.

Self-assembled and temperature-induced injectable hydrogels have been widely investigated as drug delivery systems (DDS) (Lei et al., 2017; Zhuang et al., 2019; Chen et al., 2020; Hoang Thi et al., 2020; Yang et al., 2020). The poly(lactic acid-co-glycolic acid)–poly(ethylene glycol)–poly(lactic acid-co-glycolic acid) (PLGA-PEG-PLGA) triblock copolymer, a kind of temperature-induced injectable hydrogel, with good biodegradation and biocompatibility (Yu et al., 2010; Cho et al., 2016), has been applied in many areas such as intravitreal injection (Zhang et al., 2015), subconjunctival injection (Chan et al., 2019), subcutaneous injection (Chen et al., 2016, 2016) and other local drug delivery (Bao et al., 2017; Lin et al., 2017; Thambi et al., 2017; Chen et al., 2019). When the temperature is below 37 °C, it is an aqueous solution. However, when the temperature rises to 37 °C, the injectable hydrogels convert into the gel state in situ and releases the drug slowly.

Hydrogels have been a hot spot in the research of DDS. Various studies have been recently reported about hydrogels injected into the intrathecal space or rat spinal cords (Ho et al., 2019; Householder et al., 2019; Song et al., 2019). It has been demonstrated that biodegradable PLGA pellets can effectively release drug and do not require surgical removal post drug release (Wilems & Sakiyama-Elbert, 2015). Yuanfei Wang et al. (2013), encapsulated PEGylated epidermal growth factor (PEG-EGF) in PLGA nanoparticles and erythropoietin (EPO) in PLGA-poly (sebacic acid) core-shell biphasic microparticles. The dispersion of EGF-PEG and EPO polymeric particles in a hyaluronan/methyl cellulose (HAMC) hydrogel that sustained the release of EPO for one week and EGF for three weeks continuously and inhibited the inflammatory reactions of brain tissues (Wang et al., 2013). Nano-polymer materials are expected to replace traditional drug delivery systems as a more promising approach to continuous intrathecal administration.

For the above, we propose that PLGA-PEG-PLGA triblock copolymers can be used to load AMB and inject into subarachnoid space by lumbar puncture. In this way, PLGA-PEG-PLGA triblock copolymers served as a DDS for the continuous release of AMB in CSF. In this research, we first synthesized PLGA-PEG-PLGA triblock polymers and determined their properties. The synthesized triblock polymers were then used for obtaining AMB-loaded thermogel. Further, we assessed the rheology, *in vitro* cumulative release, and its neurotoxicity, and the therapeutic effect in a rat model of CM.

## 2. Material and methods

### 2.1. Materials and animals

Polyethylene glycol (PEG, MW: 1500), stannous octoate (Sn(Oct)_2_, 95%), AMB, and AMB soluble were procured from Sigma–Aldrich. D, L-Lactide (LA), and glycolide (GA) were bought from Purac. Purified deionized water was obtained via Milli-Q plus system from Millipore (Bedford, USA). *C. neoformans* reference strain H99 (serotype A) was kindly donated by Dr.John Perfect lab. Latex-crytococcus antigen detection system was provided by IMMY (USA). Acetonitrile (ACN) was of HPLC grade, while all other chemicals were of analytical grade.

Male Sprague Dawley rats having 250–300 g bodyweight were provided by the Naval Medical University Laboratory Animal Center (Shanghai, China) and were nourished in our specific pathogen-free (SPF) animal facility. The *in vivo* experimental procedures were performed according to the national guidelines and the approval was provided by the Animal Care and Use Committee at Institute Pasteur of Shanghai.

### 2.2. Synthesis and characterization of PLGA-PEG-PLGA copolymer

PLGA-PEG-PLGA triblock copolymer was synthesized via ring-opening polymerization of GA and LA with PEG as the macroinitiator and Sn(Oct)_2_ as the catalyst (Yu et al., 2009, 2013). PEG 1500 (20.0 g) was placed in a three-necked flask and the remaining water of polymer was removed at 130 °C under vacuum for 3 h followed by cooling to 100 °C by passing argon. Then, LA (35.3 g) and GA (11.3 g) were added and the mixture was stirred well. A toluene solution containing 60 mg of Sn(Oct)_2_ was then added to the mixture. The removal of toluene was carried out under vacuum for 15 min, and the reaction was carried out at 150 °C for 12 h in the presence of argon. When the reaction was complete, the flask was kept at 110 °C for the removal of unreacted monomers (for 3 h). In the end, the resultant copolymer was washed thrice with deionized water at 80 °C followed by freeze-drying for the removal of residual water.

#### 2.2.1. ^1^h-NMR measurement

Structural elucidation and composition of the synthesized polymer were carried out with ^1^H-NMR (Bruker BioSpin International, AVANCE III HD 400 MHz) (Yu et al., 2011). Tetramethylsilane (TMS) and CDCl3 were used as the internal standard and the solvent, respectively.

#### 2.2.2. Gel permeation chromatography characterization

A gel permeation chromatography (GPC) system (Agilent 1100) was employed to identify the MWs and the distributions of the PLGA-PEG-PLGA triblock copolymers (Yu et al., 2011). The measurements were conducted at 35 °C, using tetrahydrofuran as a solvent with a flow rate of 1.0 mL/min. Monodispersed polystyrene was utilized as a standard for the calculation of MWs.

### 2.3. Characterization of sol-gel transition

#### 2.3.1. Determination of sol-gel transition temperature

The sol-gel transition temperature of the polymer aqueous solutions was measured by an inverted rotation method (Li et al., 2013). A series of polymer solutions of different concentrations were prepared. The polymeric solution (0.5 mL) was transferred into a test tube (2 mL), followed by immersing in a water bath at 24 °C. When the temperature was elevated by 1 °C, the test tubes were inverted by 180° to examine the flow of liquid. When the visible flow was not observed within 30 s, the sample was regarded as a gel state. The above-inverted rotation method was repeated until the sol-gel phase transition occurred in all concentrations of the sample.

#### 2.3.2. Rheological analysis

The rheological behavior of copolymer aqueous solution (25 wt%) and AMB-loaded copolymer aqueous solution (2, 4, and 8 mg/mL) with temperature was measured by rotational rheometer (Malvern, Kinexus Pro). The lamina acted as a clamp for samples (cone angle was 1 degree, the diameter was 60 mm) while keeping the clearance as 0.03 mm, angular velocity as 10 rad/s, the rate of heating as 0.5-degree C/min, and the temperature range as 15–50 °C (Yu et al., 2009). To prevent moisture volatilization during heating, silicone oil was evenly applied to the periphery of the fixture.

### 2.4. *In vitro* release of drug

Ultraviolet spectrophotometry was used to measure the cumulative release rate of AMB in gel with different concentrations *in vitro*. AMB-loaded copolymer aqueous solutions (2, 4, and 8 mg/mL, 0.5 mL of each one) were placed at the bottom of the glass tube, and 3 groups of parallel samples were set for each concentration. The temperature of the water bath was maintained at 37 °C. Post transformation of AMB-loaded polymer solution into the hydrogel, 10 mL of PBS having SDS (1%) and tween (0.5%) were added into a glass tube as the releasing medium (pH 7.4). All sample tubes (9 groups in total) were taken out of slow-release media (10 mL) at a specific time point and added 10 mL of fresh PBS solution at the same temperature. The drug residue in the release media was determined by an ultraviolet spectrophotometer. Samples were collected at 1 d, 2 d, 3 d, 4 d, 6 d, 8 d, 10 d, 12 d, 14 d, 16 d, 18 d, 21 d, 24 d, 27 d, 30 d, 33 d, and 36 d. The whole process was carried out under closed light conditions.

The release data was evaluated by the first-order release curve, whose equation is ln(1-M_t_/M_∞_) = -Kt (Xiao et al., 2014; Yang et al., 2014; Xie et al., 2015). Here, M_t_ is the cumulative release at time t and M_∞_ is the cumulative release at time infinity. The release rate constant k is associated with the diffusion coefficient, chemical structure of hydrogel, and some geometric parameters.

### 2.5. Toxicology evaluation

The rats were intrathecally injected with AMB in the gel to assess their toxicology through Tarlov’s scores (Tarlov, 1972) and survival rate in rats. All rats were randomized into four groups (eighteen rats in both the thermogel group and solution group and eight rats in the blank gel and NS group). The rats were anesthetized with pentobarbital (40 mg/kg) by intraperitoneal injection. For AMB in the gel group, an aliquot of 100 uL AMB-loaded thermogel (8 mg/mL) was injected into the subarachnoid space. The syringe was held for about 30 s to allow the solution to form a semi-solid gel and make sure no CSF bleeding occurred during the procedure. For the AMB solution group, 100 ul of 8 mg/mL AMB solution was injected into the subarachnoid space and the syringe held for about 30 s. The same volume of blank gel and NS was injected into the subarachnoid space for the blank gel and NS group, respectively. All the formulations were sterilized by filtration before administration. The vital signs were observed and every limb’s motor activity was evaluated after administration by the modified Tarlov’s scores (Tarlov, 1972; Shi et al., 2007): 0, complete paralysis or death; 1, muscle tension, free movement, not weight-bearing; 2, affected limbs can be weight-bearing and standing; 3, can stand normally, may limp, when walking, the limb has tow; 4, normal muscle strength. The scores of each limb were added and the total scores were recorded. On the 7th day after administration, all the rats were sacrificed and the spinal cord near the injection site was cut. The variations in spinal cord nerves were observed via Hematoxy-Eosin (HE) staining.

### 2.6. Treatment effect

The therapeutic effects were evaluated by detecting the number of cryptococcus and latex agglutination test (LAT) in CSF. A rat model of cryptococcal meningitis was established (Najvar et al., 1999; Fries et al., 2005; Carroll et al., 2007). *C. neoformans* reference strain H99 (serotype A) was recovered from −80 °C. A concentration of 2 × 10^8^ CFU/mL bacteria suspension was prepared, and 50 uL of the bacteria suspension was injected into intracranial to build cryptococcal meningitis rat model. The same volume of CSF was taken before injection for maintaining intracranial pressure (Fries et al., 2005). All rats were grouped into four randomized sets (six rats in each group). One group was a sham operation group. A cryptococcal meningitis rat model was established for the other three groups. On the 7th day after inoculation, 100 uL of AMB-load thermogel (8 mg/mL) was injected into the subarachnoid space for AMB in the gel group and 100 uL of blank thermogel were injected into the subarachnoid for the blank gel group. For the AMB solution group, 100 uL of 0.01 mg per mL, 0.02 mg per mL, 0.03 mg per mL, and 0.04 mg per mL AMB solutions were injected intrathecally at 7, 11, 15, and 19 days after infection to simulate the clinical treatment regimen. On 14 and 21 days, 100 ul CSF was taken for CSF culture and a LAT was carried out to compare treatment effects.

### 2.7. Statistical analysis

The obtained results were represented as mean ± SD. The survival rate was measured by the Log-rank test. T-test was used for samples conforming to the normal distribution. A non-parametric test was employed for samples not conforming to the normal distribution, and *p*-value < .05 revealed a statistical difference.

## 3. Results

### 3.1. Synthesis and characterization of PLGA-PEG-PLGA triblock copolymer

**Figure 1.**
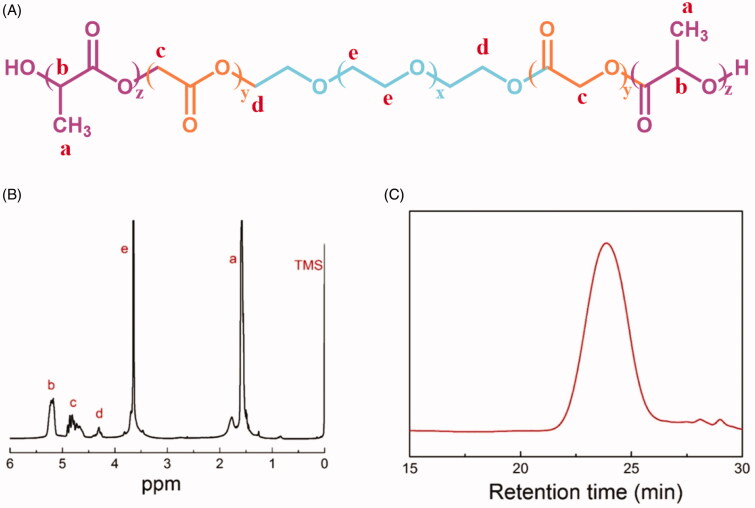
Characterization of PLGA–PEG–PLGA triblock copolymer. (A) The structure of PLGA-PEG-PLGA triblock copolymer. (B) The ^1^H NMR spectrum of PLGA–PEG–PLGA triblock copolymer in CDCl3. (C) GPC spectra of PLGA–PEG–PLGA triblock copolymer.

PLGA–PEG–PLGA triblock copolymer was synthesized by ring-opening copolymerization of LA and GA using PEG as the macroinitiator in the presence of Sn(Oct)_2_. The chemical structure of the triblock polymer is shown in Figure 1(A). ^1^H NMR spectrum of the polymer is depicted in Figure 1(B). In the LA unit, the characteristic peaks of the methyl and methine protons were at 1.55 and 5.20 ppm, accordingly while in the GA and PEG units, the characteristic peaks of methylene protons were at 4.80 and 3.60 ppm, accordingly. At 4.31 ppm, the peak of the methylene protons of the PEG segment (nearby PLGA blocks) was found. The integral peaks at 5.20, 4.80, and 3.60 ppm indicated the number-average MW (M_n_), LA/GA molar ratio, and PEG/PLGA molar ratio, respectively which had been described in the previous studies (Yu et al., 2007, 2009, 2011). The MW of the copolymers was approximately 5000. As shown in Figure 1(C), the GPC trace revealed a unimodal distribution and the dispersity was 1.28, indicating the polymerization was under control and the ideal polymer was synthesized. Molecular parameters of the copolymer determined by ^1^H NMR and GPC are indicated in Table 1.

**Table 1.** Characterization of PLGA–PEG–PLGA triblock copolymer.

Sample	M_n_^a^	PEG M_n_^b^	LA/GA (mol/mol)^a^	M_n_^c^	(M_w_/M_n_)^c^
PLGA-PEG-PLGA	1790-1500-1790	1500	2.2	5720	1.28

^a^Mn of PLGA blocks was calculated via ^1^H NMR. ^b^Mn of PEG block was provided by Aldrich. ^c^Measured by GPC.

### 3.2. Sol-gel transition of the aqueous solutions of the PLGA-PEG-PLGA triblock copolymer

**Figure 2.**
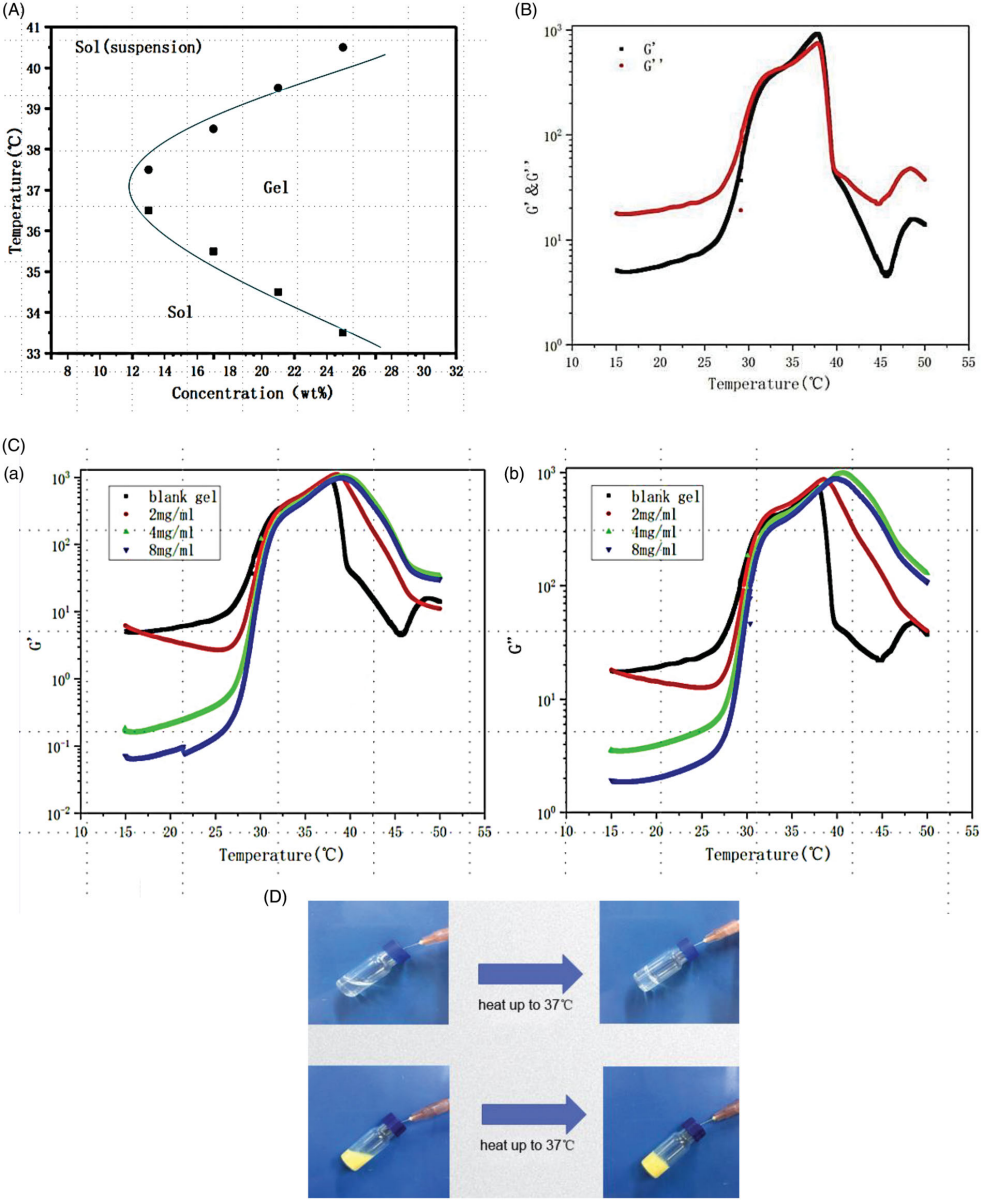
Characterization of sol-gel transition. (A) Phase diagram of PLGA–PEG–PLGA triblock copolymer aqueous solution (25 wt%). (B) The storage modulus G′ and loss modulus G″ of copolymer aqueous solution (25 wt%) as a function of temperature. (C) The storage modulus G′ and loss modulus G″ of copolymer aqueous solution (25 wt%) as a function of temperature with different AMB loading. (D) The change of blank gel (above) and AMB in the gel (below) with the temperature rise from room temperature to 37 °C.

The phase diagram of the systems is shown in Figure 2(A). The systems went through three stages with the increasing temperature: first formed free-flowing sols state, then transformed to a semi-solid gel state, and finally went to suspension. The critical gelation concentration (CGC) of the systems was about 12 wt%. The phase transition temperature of 25 wt% polymer aqueous solution was around 33.5 °C. It means that when the temperature is below 33.5 °C, the 25 wt% polymer aqueous solution is in a solution state and can be used for injection and drug loading. When the temperature is above 33.5 °C and is lower than 40.5 °C, it is in a gel state and can be used for sustained-release of drugs. The human body’s temperature is about 37 °C. Therefore, we selected 25 wt% polymer aqueous solution as the drug loading system.

As shown in Figure 2(B), the polymer aqueous solution has a low modulus at low temperature, indicating that the system has good injectability. As the temperature increases, the storage modulus G′ and the loss modulus G″ rise sharply near the phase transition temperature, indicating that the system forms a gel. According to the intersection of energy storage modulus and loss modulus as the definition of the sol-gel transition temperature, it can be determined that the gel-forming temperature is about 34 °C (Luan et al., 2018), which is close to the result of the phase diagram. As shown in Figure 2(C), the storage modulus G′ and the loss modulus G″ did not change significantly in blank gel and AMB in gel, and the sol-gel transition temperature is still the same. This means that the AMB did not affect the thermogel. Figure 2(D) shows the visual changes of the blank gel and AMB in the gel as the temperature rises to 37 °C.

### 3.3. *In-vitro* drug release

**Figure 3.**
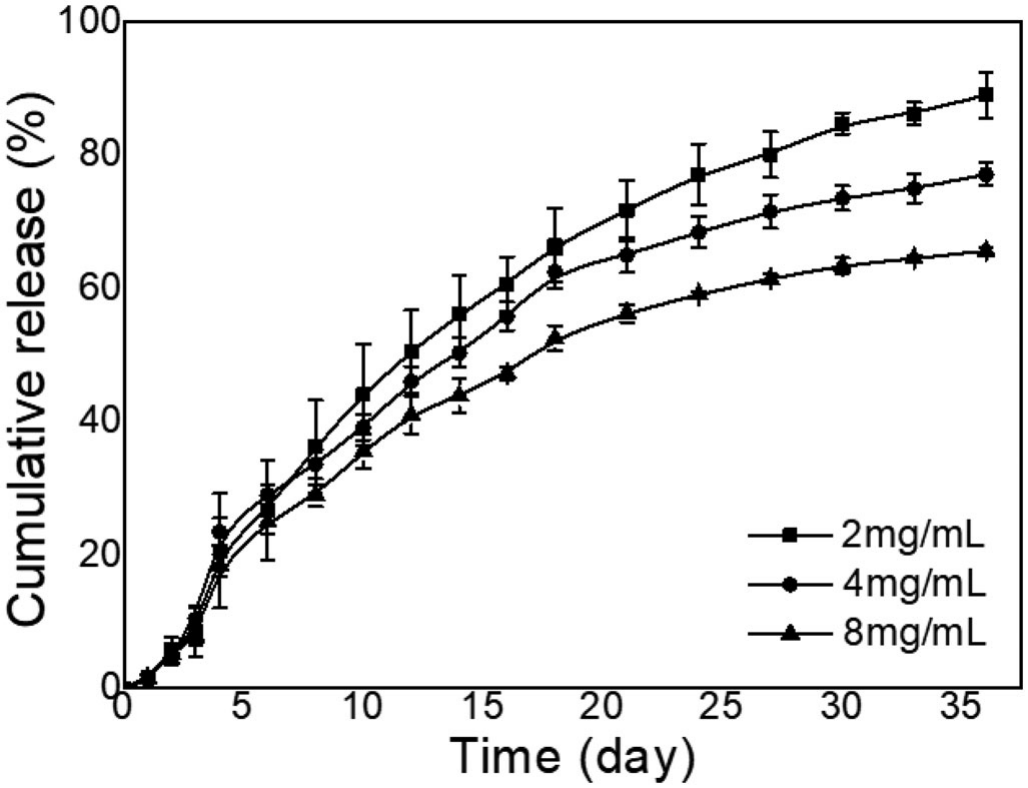
Cumulative release profiles of AMB in gel with specific concentrations in PBS at 37 °C. The concentration of the polymer was kept at 25 wt% (*n* = 3).

Figure 3 shows the cumulative release curve of AMB in gel with different AMB-loaded concentrations. On the first day, the sudden release of AMB in the gel at 2 mg per mL, 4 mg per mL, and 8 mg per mL was 1.6%, 1.3%, and 1.5%, respectively, while the cumulative release at 36 days was 89.0%, 77.1%, and 65.6%, respectively. It can be suggested that the loading concentration has little effect on the sudden release of drugs. But with elevation in the loading concentration, the cumulative release was lowered. The AMB in gel continued to release the drug slowly for 36 days. The first-order fitting results of AMB slow-release data are shown in Table 2. The three curves are consistent with the equation, and the correlation coefficient R^2^ has been greater than 0.95. This property indicates that the diffusion of the drug and the degradation of the gel are the main reasons for the slow release.

**Table 2.** First-order kinetic assessment of the in vitro release data.

AMB concentration (mg/mL)	K	M_∞_	R^2^
2	0.0567	102.7	0.9985
4	0.0652	85.9	0.9877
8	0.0679	72.4	0.9944

### 3.4. Toxicology evaluation

The Tarlov’s scores of the four groups of rats are shown in Table 3. All the rats had normal limb movements before administration. The Tarlov’s scores of the limbs of rats in the blank gel group and the NS group were 16 ± 0 points each day. The daily Tarlov’s scores of the limbs of rats in AMB in the gel group were found to be elevated than those in the AMB solution group (*p* < .05), revealing that the toxicity of AMB in the gel to the spinal cord was less than that of the AMB solution.

**Table 3.** Tarlov’s score within 7 days after intrathecal administration in rats.

Day	Blank gel (n = 8)	NS (n = 8)	AMB solution (n = 18)	AMB in gel (n = 18)	*p* ^a^
Before dosing	16 ± 0	16 ± 0	16 ± 0	16 ± 0	<.05
Day 1	16 ± 0	16 ± 0	6.11 ± 6.01	14.06 ± 3.04	<.05
Day 2	16 ± 0	16 ± 0	5.50 ± 6.11	15.61 ± 0.78	<.05
Day 3	16 ± 0	16 ± 0	5.56 ± 6.31	16 ± 0	<.05
Day 4	16 ± 0	16 ± 0	4.67 ± 6.47	16 ± 0	<.05
Day 5	16 ± 0	16 ± 0	4.89 ± 6.66	15.11 ± 3.77	<.05
Day 6	16 ± 0	16 ± 0	4.61 ± 6.94	15.11 ± 3.77	<.05
Day 7	16 ± 0	16 ± 0	4.28 ± 7.11	15.11 ± 3.77	<.05

^a^*p* values were the result of Mann-Whitney U test in AMB solution group and AMB in gel group.

**Figure 4.**
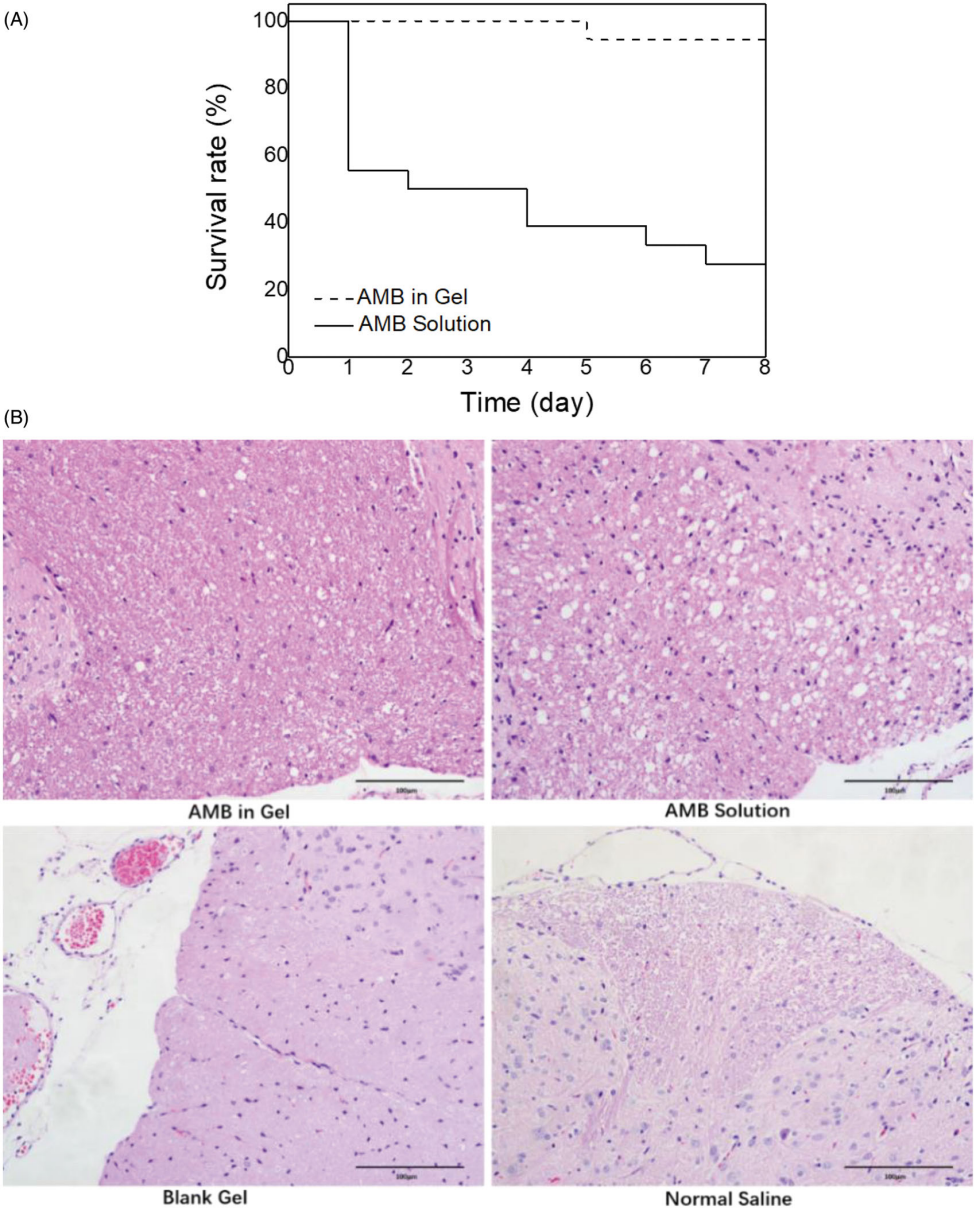
Toxicology study in rats. (A) The survival rate of intrathecal injection of AMB in gel and AMB solution in rats. (*p* < .05; *n* = 18) (B) H & E staining of the spinal cord on the 7th day after intrathecal injection of AMB in gel, AMB solution, blank gel, and Normal Saline.

The survival rate of the rats was shown in Figure 4(A). There was no death in the blank gel group and the NS group, and the 7-day survival rate was 100%. In the AMB in the gel group, 1 rat died on the 5th day after administration, and the 7-day survival rate was 94.4%. Eight rats in the AMB solution group died on the day of administration, followed by death every 1-2 days, with a 7-day survival rate of 27.8%. The survival rate of the AMB in the gel group was significantly elevated relative to the AMB solution group (*p* < .05), indicating that thermogel could reduce the mortality caused by neurotoxicity.

The H&E staining of the spinal cord exposed to different groups is shown in Figure 4(B). AMB solution group showed that the injury site was mainly in the posterior cord of white matter. The boundary between gray and white matter was found to be not clear, some nerve fibers dissolved and disappeared, and the loose tissues underwent edema, while some cells showed vacuolar degeneration and karyopyknosis. The AMB in the gel group exhibited a clear boundary between gray and white matter, a small amount of fiber disorder, and cellular vacuolar degeneration. NS and blank gel group exhibited the regular morphology.

### 3.5. Treatment effect

**Figure 5.**
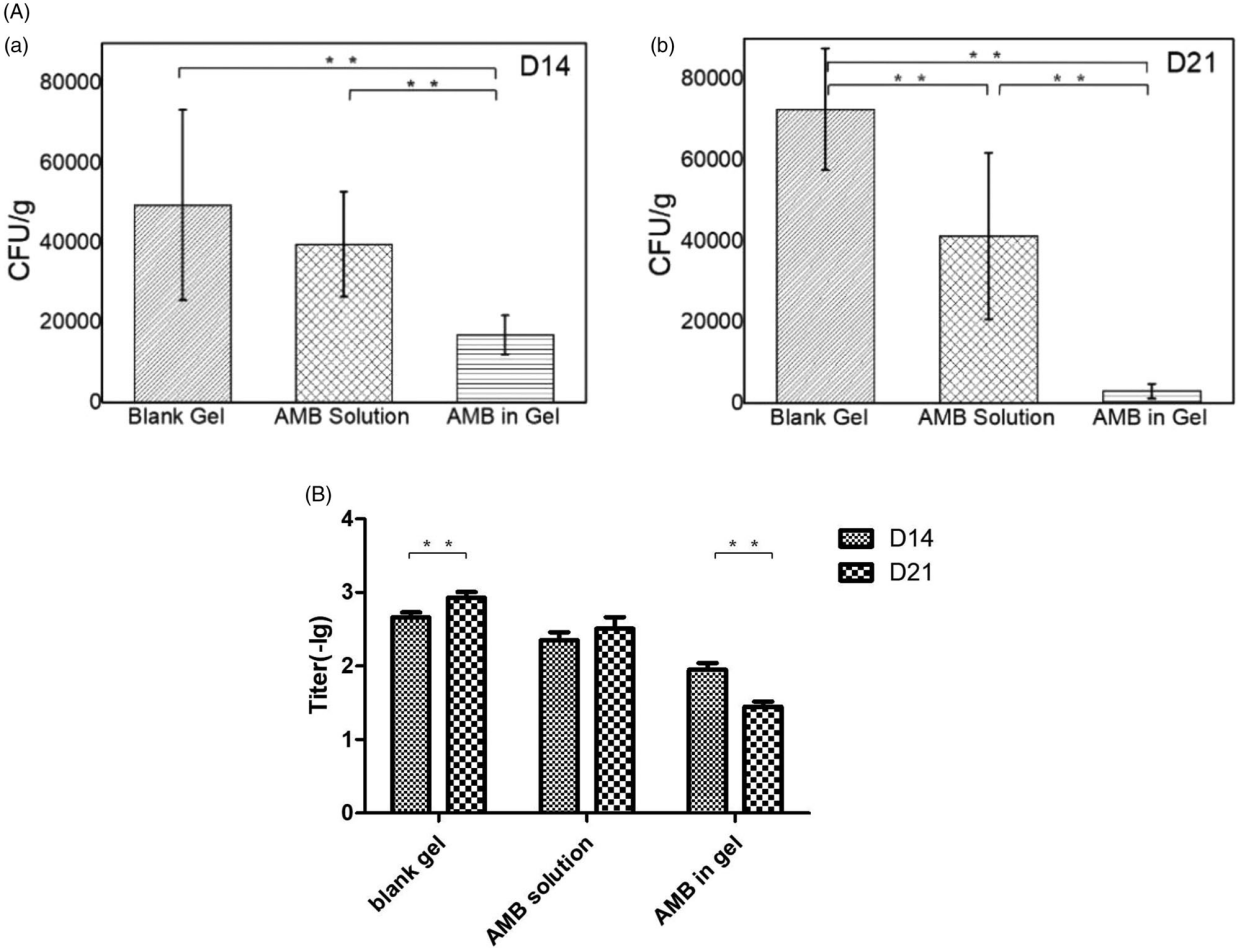
Therapeutic effect in rats. (a) The count of CSF on the 14th day after modeling. (b) The count of cerebrospinal fluid culture on the 21st day after modeling. (B) Latex agglutination test of CSF on the 14th and 21st days after modeling. **p < .05; (*n* = 6).

Figure 5(A) depicts the results of CSF culture on the 14th and 21st days after rat modeling, respectively. On day 14, the bacterial loads of the blank gel group, the AMB solution group, and the AMB in the gel group were found to be 49,500 ± 23,855 CFU/mL, 39,667 ± 12,140 CFU/mL, and 17,000 ± 48,757 CFU/mL, respectively. On day 21, the bacterial loads of the blank gel group, the AMB solution group, and the AMB in the gel group were found to be 72,600 ± 15,059 CFU/mL, 41,333 ± 20,490 CFU/mL, and 3000 ± 1786 CFU/mL, respectively.

The LAT is a sensitive indicator to detect the antigenic titer of the cryptococcus polysaccharide capsule. As evident from Figure 5(B), from day 14 to day 21, the average titer of the blank gel increased from 1:453 to 1:844 (*p* < .05), the average titer of the AMB solution increased from 1:22.6 to 1:32 (*p* > .05), and the average titer of the AMB in gel decreased from 1:89 to 1:28 (*p* < .05). It can be seen from the figures that the results of LAT were consistent with the results of CSF culture in both longitudinal and lateral comparisons, which verified that the therapeutic effect of AMB in the gel was superior to that of AMB solution again.

## Discussion

AMB is an irreplaceable drug for the treatment of CM, however, patients mostly discontinue the medication because they are unable to tolerate its side effects. There are many studies aimed at improving AMB efficacy and reducing its toxicity (Zumbuehl et al., 2007; Hudson et al., 2010; Asthana et al., 2015; Kumar et al., 2015; Sosa et al., 2017), however, they have not been advantageous over the thermosensitive PLGA-PEG-PLGA hydrogel. Firstly, the structure and properties of thermosensitive PLGA-PEG-PLGA hydrogel are more suitable for loading hydrophobic drugs. AMB is extremely difficult to dissolve in water and can be well wrapped in a hydrophobic region with nearly 100% entrapment efficiency. Secondly, the PLGA-PEG-PLGA hydrogel has good biocompatibility. Its degradation products have already been present in the body and can be completely decomposed and consumed. Thirdly, temperature-sensitive properties allow PLGA-PEG-PLGA hydrogel to be used for injection, and the ideal concentration of AMB can be continuously provided in areas where the fungal infection is serious. Fourthly, the controlled-release characteristics can reduce the dosing frequency. Continuous and ideal drug concentration can kill Cryptococcus, thus leading to effective treatment of the disease. Finally, neurotoxicity associated with AMB high concentration causes irreversible damage to spinal nerves. Intrathecal injection of AMB-loaded thermogel can avoid high AMB concentration and reduce neurotoxicity. All the above properties make AMB-loaded thermogel suitable for intrathecal injection of CM.

PLGA-PEG-PLGA triblock copolymers have been extensively studied for the delivery of drugs. Liraglutide, granulocyte colony-stimulating factor, porcine growth hormone, recombinant hepatitis B surface antigen, and antitumor drugs have been loaded in the thermogel (Matthes et al., 2007; DuVall et al., 2009; Elstad & Fowers, 2009; Tyler et al., 2010; Chen et al., 2016; Cho et al., 2016). In our study, a thermoreversible hydrogel comprising of copolymers with different features was prepared as an injectable implant for intrathecal DDS. The PLGA–PEG–PLGA triblock copolymer was synthesized and underwent a reversible sol-gel transition as the temperature elevated. The results showed that the synthesis, structural control, and molecular weight were within expectations (Yu et al., 2009, 2011, 2013). For the preparation of injectable AMB-loaded thermogel, AMB was loaded into the aqueous polymer solution at 4 °C, followed by stirring to obtain a homogeneous system. From the microscopic point of view, PLGA-PEG-PLGA is a triblock polymer. The PLGA at both ends of the polymer is hydrophobic and the PEG in the middle is a hydrophilic region, which makes the polymer spontaneously assembled into spherical particles upon contact with an aqueous environment (Yu et al., 2007, 2009). AMB is a hydrophobic drug and can be entrapped into the hydrophobic region of the thermogel. When the temperature rises to sol-gel transition temperature (33.5 °C), the particles interact with each other, thus form a network and become a gel state. This in turn provides increased loading of the AMB in the thermogel.

The result of *in vitro* release showed that a small part of AMB failed in the process. The higher loading concentration resulted in a higher rate of failure. The factors affecting AMB failure are not only its instability and loading concentration but also temperature and light. However, the AMB solubility has been very low in the water, little AMB is still dissolved in water. The main reason for the sudden release of AMB in the gel is the release of AMB which is dissolved in water. When the thermogel slowly degrades in an aqueous solution, the AMB wrapped in the hydrophobic region is exposed. The AMB concentration in the hydrophobic region is high, while it is very low in the solution. So, AMB will diffuse from high concentration to low concentration, thus it is slowly released (Yu et al., 2008). The gel can release its loaded AMB for 36 days *in vitro*. PLGA-PEG-PLGA triblock copolymer degrades into ethanol, lactic acid, and glycolic acid, thus it has good biocompatibility.

In the toxicity study of the Tarlov’s score, all surviving rats had normal upper limbs. Most of the dead rats had respiratory arrest within 2–3 minutes after administration, followed by cyanosis, and eventually suffocated. The probable reason was that AMB solution diffused from the level of the lower lumbar spine to the thoracic vertebrae and cervical spine, damaged respiratory motor neurons, caused apnea and ultimately lead to suffocation. However, when the AMB-loaded thermogel was injected, it turned into a gel state and remained in situ. So, AMB could not diffuse nonspecifically. According to the in vitro release curve, the burst release of 8 mg/mL AMB gel on the first day was 12 ug, much less than that of AMB solution of 800 ug. It can be seen from the above discussion that sustained release and in situ immobilization were the main reasons for reduced neurotoxicity and the high survival rate of AMB in the gel. The result of H&E staining also showed that the AMB in gel could reduce the neurotoxicity of AMB and blank gel resulted in no damage to the spinal cord.

In the pre-experiment, 100 uL of the AMB solution with a concentration of 2 mg per mL was used for intrathecal administration twice a week, four times in total. Most of the rats died on the day after the first administration, and all of the rats died after the second administration. Therefore, it was hard to set up the AMB solution group (2 mg per mL, 100 uL, 4 times) with the same dosage as the AMB-loaded thermogel (8 mg per mL, 100 uL, once) for pharmacodynamic comparison. Clinically, the AMB solution is injected intrathecally 2–3 times a week according to the patient's condition, starting from 1 mg and increasing by 1 mg each time until the patient is intolerant and maintaining this dose. In this study, according to the clinical administration mode and the volume ratio of CSF between humans and rats, we added the corresponding dose of soluble AMB to the CSF of rats to simulate the clinical treatment as the control group. It can be seen from the results that the AMB solution could keep the number of cryptococcus from increasing, while AMB in the gel can significantly reduce the number and titer of cryptococcus.

Our study has several limitations. Firstly, although rats showed a higher survival rate in AMB in the gel group, one rat died on the 5th day after administration. Therefore, the substantial toxicity of PLGA-PEG-PLGA triblock copolymers could not be ruled out. Secondly, toxicity was evaluated by the modified Tarlov’s scores which were mainly based on observation. Thus, the results of the toxicity experiment can be biased. Thirdly, the cumulative release of the drug in vivo may be different from that in vitro, therefore further in vivo investigation is required on pharmacokinetics and therapeutic effect.

Taken together, as compared to the traditional treatment regimen, AMB-loaded thermal was found to be convenient, and more effective with less neurotoxicity. The PLGA-PEG-PLGA triblock copolymers are biocompatible and can effectively deliver an intrathecal drug. Currently, intrathecal hydrogels are widely reported in the repair of spinal cord injury. Dongfei Liu et al. (2016), reported an in-situ gelling drug delivery system, comprising a Poloxamer-407, a 188 mixture-based thermoresponsive hydrogel matrix and, an incorporated bioactive compound (monosialoganglioside, GM1), for spinal cord injury therapy. The thermoresponsive hydrogel delayed the GM1 release for around one month. Irja Elliott Donaghue et al. (2015), reported HAMC hydrogel, an injectable and biodegradable polymeric nanoparticle, as an injection into the intrathecal space for acute local delivery. Neurotrophin-3 was encapsulated in the hydrogel and its in vivo release was observed for 28 d.

AMB plays an important role in the treatment of CM. It is of great significance to increase the concentration of AMB in CSF for CM. Intrathecal injection of AMB-loaded hydrogel can not only enhance the concentration of AMB in CSF but also greatly reduce the neurotoxicity of AMB. Its sustained-release can minimize dosing frequency and improve patient compliance, thus suggesting the hydrogel as an ideal drug delivery system for the treatment of CM. CM is hard to be treated effectively due to its high recurrence rate and unsatisfactory therapeutic efficacy. The AMB-loaded hydrogel is a breakthrough in the treatment of CM. In this study, CM was used as a model to explore the new route of intrathecal administration.

## Conclusion

In the present study, an intrathecal DDS was developed for the treatment of CM. Thermosensitive injectable PLGA-PEG-PLGA hydrogel was fabricated based on a practical blending approach and was then employed to deliver AMB. The AMB-loaded thermogel could release AMB for 36 days *in vitro* with a high cumulative release rate. The *in vivo* studies showed that intrathecal injection of AMB in gel could reduce its dosing frequency and associated neurotoxicity, and improve the antifungal efficiency. Intrathecal administration of AMB in the gel is a very effective antifungal therapy for the CNS, which provides a good reference for intrathecal drug delivery.

## References

[CIT0001] Asthana S, Jaiswal AK, Gupta PK, et al. (2015). Th-1 biased immunomodulation and synergistic antileishmanial activity of stable cationic lipid-polymer hybrid nanoparticle: biodistribution and toxicity assessment of encapsulated amphotericin B. Eur J Pharm Biopharm 89:62–73. 25477079 10.1016/j.ejpb.2014.11.019

[CIT0002] Bao X, Zhu L, Huang X, et al. (2017). 3D biomimetic artificial bone scaffolds with dual-cytokines spatiotemporal delivery for large weight-bearing bone defect repair. Sci Rep 7:7814. 28798376 10.1038/s41598-017-08412-0 PMC5552682

[CIT0003] Carroll SF, Guillot L, Qureshi ST. (2007). Mammalian model hosts of cryptococcal infection. Comp Med 57:9–17. 17348287

[CIT0004] Chan PS, Xian JW, Li Q, et al. (2019). Biodegradable Thermosensitive PLGA-PEG-PLGA Polymer for Non-irritating and Sustained Ophthalmic Drug Delivery. Aaps J 21:59. 31020458 10.1208/s12248-019-0326-x

[CIT0005] Chen X, Wang M, Yang X, et al. (2019). Injectable hydrogels for the sustained delivery of a HER2-targeted antibody for preventing local relapse of HER2+ breast cancer after breast-conserving surgery. Theranostics 9:6080–98. 31534538 10.7150/thno.36514 PMC6735507

[CIT0006] Chen X, Zhang J, Wu K, et al. (2020). Visualizing the *in vivo* evolution of an injectable and thermosensitive hydrogel using tri-modal bioimaging. Small Methods 4:2000310.

[CIT0007] Chen Y, Li Y, Shen W, et al. (2016). Controlled release of liraglutide using thermogelling polymers in treatment of diabetes. Sci Rep 6:31593. 27531588 10.1038/srep31593 PMC4987673

[CIT0008] Chen Y, Luan J, Shen W, et al. (2016). Injectable and thermosensitive hydrogel containing liraglutide as a long-acting antidiabetic system. ACS Appl Mater Interfaces 8:30703–13. 27786459 10.1021/acsami.6b09415

[CIT0009] Cho H, Gao J, Kwon GS. (2016). PEG-b-PLA micelles and PLGA-b-PEG-b-PLGA sol-gels for drug delivery. J Control Release 240:191–201. 26699425 10.1016/j.jconrel.2015.12.015 PMC4909590

[CIT0010] DuVall GA, Tarabar D, Seidel RH, et al. (2009). Phase 2: a dose-escalation study of OncoGel (ReGel/paclitaxel), a controlled-release formulation of paclitaxel, as adjunctive local therapy to external-beam radiation in patients with inoperable esophageal cancer. Anticancer Drugs 20:89–95. 19209024 10.1097/CAD.0b013e3283222c12

[CIT0011] Elliott Donaghue I, Tator CH, Shoichet MS. (2015). Sustained delivery of bioactive neurotrophin-3 to the injured spinal cord. Biomater Sci 3:65–72. 26214190 10.1039/c4bm00311j

[CIT0012] Elstad NL, Fowers KD. (2009). OncoGel (ReGel/paclitaxel)-clinical applications for a novel paclitaxel delivery system. Adv Drug Deliv Rev 61:785–94. 19422870 10.1016/j.addr.2009.04.010

[CIT0013] Fang M, Lü TM, Ma AD, et al. (2012). Comparative pharmacokinetics of continuous and conventional intrathecal amphotericin B in rabbits. Antimicrob Agents Chemother 56:5253–7. 22850516 10.1128/AAC.00304-12 PMC3457388

[CIT0014] Fries BC, Lee SC, Kennan R, et al. (2005). Phenotypic switching of Cryptococcus neoformans can produce variants that elicit increased intracranial pressure in a rat model of cryptococcal meningoencephalitis. Infect Immun 73:1779–87. 15731079 10.1128/IAI.73.3.1779-1787.2005 PMC1064965

[CIT0015] Hamill RJ. (2013). Amphotericin B formulations: a comparative review of efficacy and toxicity. Drugs 73:919–34. 23729001 10.1007/s40265-013-0069-4

[CIT0016] Ho MT, Teal CJ, Shoichet MS. (2019). A hyaluronan/methylcellulose-based hydrogel for local cell and biomolecule delivery to the central nervous system. Brain Res Bull 148:46–54. 30898580 10.1016/j.brainresbull.2019.03.005

[CIT0017] Hoang Thi TT, Sinh LH, Huynh DP, et al. (2020). Self-assemblable polymer smart-blocks for temperature-induced injectable hydrogel in biomedical applications. Front Chem 8:19. 32083052 10.3389/fchem.2020.00019 PMC7005785

[CIT0018] Householder KT, Dharmaraj S, Sandberg DI, et al. (2019). Fate of nanoparticles in the central nervous system after intrathecal injection in healthy mice. Sci Rep 9:12587. 31467368 10.1038/s41598-019-49028-w PMC6715675

[CIT0019] Hudson SP, Langer R, Fink GR, et al. (2010). Injectable *in situ* cross-linking hydrogels for local antifungal therapy. Biomaterials 31:1444–52. 19942285 10.1016/j.biomaterials.2009.11.016 PMC2813953

[CIT0020] Kumar R, Sahoo GC, Pandey K, et al. (2015). Study the effects of PLGA-PEG encapsulated amphotericin B nanoparticle drug delivery system against Leishmania donovani. Drug Deliv 22:383–8. 24601828 10.3109/10717544.2014.891271

[CIT0021] Lei K, Chen Y, Wang J, et al. (2017). Non-invasive monitoring of *in vivo* degradation of a radiopaque thermoreversible hydrogel and its efficacy in preventing post-operative adhesions. Acta Biomater 55:396–409. 28363786 10.1016/j.actbio.2017.03.042

[CIT0022] Li K, Yu L, Liu X, et al. (2013). A long-acting formulation of a polypeptide drug exenatide in treatment of diabetes using an injectable block copolymer hydrogel. Biomaterials 34:2834–42. 23352120 10.1016/j.biomaterials.2013.01.013

[CIT0023] Lin FW, Chen PY, Wei KC, et al. (2017). Rapid *in situ* mri traceable gel-forming dual-drug delivery for synergistic therapy of brain tumor. Theranostics 7:2524–36. 28744332 10.7150/thno.19856 PMC5525754

[CIT0024] Liu D, Jiang T, Cai W, et al. (2016). An *in situ* gelling drug delivery system for improved recovery after spinal cord injury. Adv Healthc Mater 5:1513–21. 27113454 10.1002/adhm.201600055

[CIT0025] Luan J, Zhang Z, Shen W, et al. (2018). Thermogel loaded with low-dose paclitaxel as a facile coating to alleviate periprosthetic fibrous capsule formation. ACS Appl Mater Interfaces 10:30235–46. 30102023 10.1021/acsami.8b13548

[CIT0026] Matthes K, Mino-Kenudson M, Sahani DV, et al. (2007). EUS-guided injection of paclitaxel (OncoGel) provides therapeutic drug concentrations in the porcine pancreas (with video). Gastrointest Endosc 65:448–53. 17173909 10.1016/j.gie.2006.06.030

[CIT0027] Migone C, Ford N, Garner P, et al. (2018). Updating guidance for preventing and treating cryptococcal disease: how evidence and decisions interface. Cochrane Database Syst Rev 11:ED000130. 30520517 10.1002/14651858.ED000130 PMC10284627

[CIT0028] Najvar LK, Bocanegra R, Graybill JR. (1999). An alternative animal model for comparison of treatments for cryptococcal meningitis. Antimicrob Agents Chemother 43:413–4. 9925548 10.1128/aac.43.2.413 PMC89093

[CIT0029] Nakama T, Yamashita S, Hirahara T, et al. (2015). Usefulness of intraventricular infusion of antifungal drugs through Ommaya reservoirs for cryptococcal meningitis treatment. J Neurol Sci 358:259–62. 26362338 10.1016/j.jns.2015.09.005

[CIT0030] Nau R, Blei C, Eiffert H. (2020). Intrathecal Antibacterial and Antifungal Therapies. Clin Microbiol Rev 33:e00190-19. 32349999 10.1128/CMR.00190-19 PMC7194852

[CIT0031] Perfect JR, Dismukes WE, Dromer F, et al. (2010). Clinical practice guidelines for the management of cryptococcal disease: 2010 update by the infectious diseases society of america. Clin Infect Dis 50:291–322. 20047480 10.1086/649858 PMC5826644

[CIT0032] Rajasingham R, Smith RM, Park BJ, et al. (2017). Global burden of disease of HIV-associated cryptococcal meningitis: an updated analysis. Lancet Infect Dis 17:873–81. 28483415 10.1016/S1473-3099(17)30243-8 PMC5818156

[CIT0033] Shi E, Jiang X, Kazui T, et al. (2007). Controlled low-pressure perfusion at the beginning of reperfusion attenuates neurologic injury after spinal cord ischemia. J Thorac Cardiovasc Surg 133:942–8. 17382631 10.1016/j.jtcvs.2006.12.017

[CIT0034] Song YH, Agrawal NK, Griffin JM, et al. (2019). Recent advances in nanotherapeutic strategies for spinal cord injury repair. Adv Drug Deliv Rev 148:38–59. 30582938 10.1016/j.addr.2018.12.011 PMC6959132

[CIT0035] Sosa L, Clares B, Alvarado HL, et al. (2017). Amphotericin B releasing topical nanoemulsion for the treatment of candidiasis and aspergillosis. Nanomedicine 13:2303–12. 28712917 10.1016/j.nano.2017.06.021

[CIT0036] Tarlov IM. (1972). Acute spinal cord compression paralysis. J Neurosurg 36:10–20. 5007267 10.3171/jns.1972.36.1.0010

[CIT0037] Thambi T, Li Y, Lee DS. (2017). Injectable hydrogels for sustained release of therapeutic agents. J Control Release 267:57–66. 28827094 10.1016/j.jconrel.2017.08.006

[CIT0038] Tyler B, Fowers KD, Li KW, et al. (2010). A thermal gel depot for local delivery of paclitaxel to treat experimental brain tumors in rats. J Neurosurg 113:210–7. 20001591 10.3171/2009.11.JNS08162

[CIT0039] Wang Y, Cooke MJ, Sachewsky N, et al. (2013). Bioengineered sequential growth factor delivery stimulates brain tissue regeneration after stroke. J Control Release 172:1–11. 23933523 10.1016/j.jconrel.2013.07.032

[CIT0040] Wilems TS, Sakiyama-Elbert SE. (2015). Sustained dual drug delivery of anti-inhibitory molecules for treatment of spinal cord injury. J Control Release 213:103–11. 26122130 10.1016/j.jconrel.2015.06.031 PMC4691576

[CIT0041] Williamson PR, Jarvis JN, Panackal AA, et al. (2017). Cryptococcal meningitis: epidemiology, immunology, diagnosis and therapy. Nat Rev Neurol 13:13–24. 27886201 10.1038/nrneurol.2016.167

[CIT0042] Xiao Y, Fan Y, Wang W, et al. (2014). Novel GO-COO-β-CD/CA inclusion: its blood compatibility, antibacterial property and drug delivery. Drug Deliv 21:362–9. 24164542 10.3109/10717544.2013.846997

[CIT0043] Xie F, Ji S, Cheng Z. (2015). *In vitro* dissolution similarity factor (f2) and *in vivo* bioequivalence criteria, how and when do they match? Using a BCS class II drug as a simulation example. Eur J Pharm Sci 66:163–72. 25315411 10.1016/j.ejps.2014.10.002

[CIT0044] Yang M, Xie S, Li Q, et al. (2014). Effects of polyvinylpyrrolidone both as a binder and pore-former on the release of sparingly water-soluble topiramate from ethylcellulose coated pellets. Int J Pharm 465:187–96. 24530810 10.1016/j.ijpharm.2014.02.021

[CIT0045] Yang X, Chen X, Wang Y, et al. (2020). Sustained release of lipophilic gemcitabine from an injectable polymeric hydrogel for synergistically enhancing tumor chemoradiotherapy. Chem Eng J 396:125320.

[CIT0046] Yu L, Chang G, Zhang H, et al. (2007). Temperature-induced spontaneous sol-gel transitions of poly(D,L-lactic acid-co-glycolic acid)-b-poly(ethylene glycol)-b-poly(D,L-lactic acid-co-glycolic acid) triblock copolymers and their end-capped derivatives in water. J Polym Sci A Polym Chem 45:1122–33.

[CIT0047] Yu L, Chang GT, Zhang H, et al. (2008). Injectable block copolymer hydrogels for sustained release of a PEGylated drug. Int J Pharm 348:95–106. 17825508 10.1016/j.ijpharm.2007.07.026

[CIT0048] Yu L, Sheng W, Yang D, et al. (2013). Design of molecular parameters to achieve block copolymers with a powder form at dry state and a temperature-induced sol-gel transition in water without unexpected gelling prior to heating. Macromol Res 21:207–15.

[CIT0049] Yu L, Zhang Z, Ding J. (2011). Influence of LA and GA sequence in the PLGA block on the properties of thermogelling PLGA-PEG-PLGA block copolymers. Biomacromolecules 12:1290–7. 21361277 10.1021/bm101572j

[CIT0050] Yu L, Zhang Z, Zhang H, et al. (2009). Mixing a sol and a precipitate of block copolymers with different block ratios leads to an injectable hydrogel. Biomacromolecules 10:1547–53. 19385649 10.1021/bm900145g

[CIT0051] Yu L, Zhang Z, Zhang H, et al. (2010). Biodegradability and biocompatibility of thermoreversible hydrogels formed from mixing a sol and a precipitate of block copolymers in water. Biomacromolecules 11:2169–78. 20690723 10.1021/bm100549q

[CIT0052] Yuchong C, Jianghan C, Hai W, et al. (2011). Lumbar puncture drainage with intrathecal injection of amphotericin B for control of cryptococcal meningitis. Mycoses 54:e248–e251. 20070534 10.1111/j.1439-0507.2009.01847.x

[CIT0053] Zhang L, Shen W, Luan J, et al. (2015). Sustained intravitreal delivery of dexamethasone using an injectable and biodegradable thermogel. Acta Biomater 23:271–81. 26004219 10.1016/j.actbio.2015.05.005

[CIT0054] Zhuang Y, Yang X, Li Y, et al. (2019). Sustained release strategy designed for lixisenatide delivery to synchronously treat diabetes and associated complications. ACS Appl Mater Interfaces 11:29604–18. 31361112 10.1021/acsami.9b10346

[CIT0055] Zumbuehl A, Ferreira L, Kuhn D, et al. (2007). Antifungal hydrogels. Proc Natl Acad Sci USA 104:12994–8. 17664427 10.1073/pnas.0705250104 PMC1941801

